# Supplementary material for: FoRSHE-X digital health intervention to improve the quality of life during chemotherapy among gynecological cancer survivors in Indonesia: A protocol for a pilot and feasibility study
Source: PLoS One. 2024 Dec 18;19(12):e0299901. doi: 10.1371/journal.pone.0299901 (PMC11654957; doi:10.1371/journal.pone.0299901)
Supplement: S1 Protocol — (PDF) [file pone.0299901.s002.pdf]

RESEARCH PROTOCOL

DEVELOPMENT OF FORSHE-X DIGITAL INTERVENTIONS (EDUCATION AND *TELE - COACHING)* TO IMPROVE THE QUALITY OF LIFE OF GYNECOLOGICAL CANCER PATIENTS: PILOT AND FEASIBILITY STUDY

Researchers:

1. **Prof. Dr. Yati Afiyanti, S.Kp., MN**
2. **Dyah Juliastuti, M.Sc., M.Kep ., Sp.Mat , Ph.D**
3. **Ns. Ariesta Milanti , S.Kep ., MHC, Ph.D**
4. **Ns. Lina Anisa Nasution, S.Kep ., M.Kep .**
5. **Ns. Aprilia Dian Prawesti , S.Kep**

Faculty of Nursing
Universitas Indonesia
2023

Title (*Title*)
Development of Digital Intervention (Education and *Tele-coaching*) FoRSHE-X to improve the Quality of Life of Gynecological Cancer Patients: Pilot and Feasibility Study

Abstract (*Abstract)*
Gynecological cancer is a non-communicable disease that has the highest prevalence and mortality in Indonesian women. Worsening conditions due to gynecological cancer can increase medical costs and reduce the quality of life of cancer survivors. This can be prevented by providing digital technology-based nursing interventions which have been proven to improve the physical, mental and sexual health of cancer survivors in various countries. This research aims to develop feasibility and acceptability and evaluate the effect of educational package interventions digital and *tele-coaching* FoRSHE-X (*Figure on Recurring anxiety, Self-efficacy , Health Effects, and sexual deprivation)* in gynecological cancer *survivors* who are undergoing chemotherapy. The design of this pilot study is a prospective longitudinal *mixed-methods study* that evaluates the implementation process and results, as well as the feasibility of the FoRSHE-X digital intervention in a study that integrates the use of technological media , digital education, and *tele - coaching* concepts. in increasing independence gynecological cancer survivors cope with a variety of side effects chemotherapy and improving the sustainability of cancer therapy .

Keywords
self-efficacy, side effects, FoRSHE-X, gynecological cancer, anxiety, quality of sexual relations, survivors , self-care, pilot, feasibility

Background *(Background)*
The highest cause of death in women is cancer (Sung et al., 2021) . The most common types of cancer in women are breast cancer, cervical cancer, ovarian cancer and uterine corpus cancer (Sung et al., 2021) . The magnitude of these cases is of course in line with the amount of attention needed by cancer survivors, especially regarding survival rates (*survivor rates*) and the quality of life of survivors which are issues in the *Sustainable Development Goals* (SDGs) in the health sector. Treatment for survivors of this disease is included in the nine main programs launched by the Ministry of Health (Afiyanti, Gayatri, Besral, & Haryani, 2019; Afiyanti, Milanti, & Putri, 2018) .

Gynecological cancer survivors will have a better prognosis for survival (*survivor rate)* if they are able to complete the planned series of therapy and understand health management during and after therapy (Mlakar et al., 2021; Schlumbrecht, Sun, Huang, Milbourne, & Bodurka, 2018) . When undergoing therapy, gynecological cancer survivors have multidimensional needs which, if not anticipated, can result in discontinuation of therapy, recurrence, and even death (Galica, Giroux, Francis, & Maheu, 2020; Nekhlyudov et al., 2019) . These needs are related to preventing the risk of recurrence, healthy lifestyles, managing physical symptoms due to the course of the disease as well as long-term effects of therapy and psychosocial and sexual problems, as well as informational needs regarding

independent self-care (Beesley, Alemayehu, & Webb, 2018; Galica et al. al., 2022; Schlumbrecht et al., 2018) .

Several previous studies in Indonesia showed that gynecological cancer survivors reported limited time and opportunities to ask questions and discuss with the health team, especially the nursing team, regarding the physical, mental, social and sexual problems they faced while undergoing cancer therapy (Afiyanti et al., 2019; Afiyanti, Setyowati, Milanti, & Young, 2020; Boa & Grenman, 2018) . Barriers in communicating health problems and other related matters can result in stopping therapy, delays in seeking help, and the inability of female survivors to anticipate recurrence and conflict with their sexual partners (Lokich, 2019; McCallum et al., 2014) . The use of digital technology has been proven effective in improving the physical, mental and sexual health of survivors of various types of gynecological cancer in several countries (Barakat et al., 2017; Nápoles et al., 2019; Wagner et al., 2021) .

The health problems and care needs of gynecological cancer survivors have been explored by various previous research, however , internet-based nursing interventions for cancer survivors have not been intensively developed in nursing services in Indonesia and intervention methods that can reach a wider range of nursing services have not yet been found. sensitive to Indonesian culture. Utilization It is hoped that continuous tele-education and *tele-coaching* can support the self-efficacy of gynecological cancer survivors in preventing and overcoming problems that may prevent them from continuing therapy and prepare them for the post-therapy period. This intervention can connect patients with the nursing team without being limited by space and time. It is believed that the use of digital technology can expand the reach of nursing services and increase patient participation in their own care on an ongoing basis. FoRSHE-X's digital intervention has potential fulfil need survivors' health gynecological cancer who are undergoing chemotherapy and increasing resistance life that its achievements Still low in Indonesia as well as reducing the burden on families and the State due to medical costs cancer.

Aim (*Research General Aim*)

pilot study aims to develop feasibility and acceptability and evaluate the effect of the educational package intervention digital and *tele-coaching* FoRSHE-X (*Figure on Recurring anxiety, Self-efficacy , Health Effects, and sexual deprivation)* in gynecological cancer *survivors* who are undergoing chemotherapy .

Specific Research *Objectives*

1. Explore general characteristics (age, highest level of education, employment, marital status) and health history (length of cancer diagnosis, stage of cancer, type and number of cancer therapy sessions undertaken) , as well as readiness of gynecological cancer survivors who are undergoing chemotherapy to take part in activities .
2. Evaluate the implementation process and results, as well as satisfaction and challenges for gynecological cancer survivors undergoing chemotherapy in utilizing the FoRSHE-X digital intervention package.
3. Evaluating the feasibility and acceptability of the FoRSHE-X digital intervention package in improving the quality of life of gynecological cancer survivors.

Hypothesis in this research:

1. There is a decrease in the level of anxiety in gynecological cancer survivors who are undergoing chemotherapy after administering the FoRSHE-X package intervention
2. There is an increase in self-efficacy of gynecological cancer survivors who are undergoing chemotherapy. after administering the FoRSHE-X package intervention
3. There was an increase in knowledge and a decrease in the quantity of side effects in gynecological cancer survivors who were undergoing chemotherapy after administering the FoRSHE-X package intervention
4. There is greater improvement in the quality of sexual life in gynecological cancer survivors undergoing chemotherapy. after administering the FoRSHE-X package intervention

FoRSHE-X Digital Intervention

Prototive package digital (education and tele-coach) ForSHE-X is a digital intervention that combines social media-based education (Instagram and Zoom) and *tele- coaching* (via WhatsApp video call or Zoom) to support the needs of care for gynecological cancer survivors that is sensitive to Indonesian culture a. The interventions in the FoRSHE-X package are focused on reducing anxiety about the therapy process , increasing knowledge and quantity of side effects , increasing self-efficacy, and improving the quality of sexual life of gynecological cancer survivors while undergoing a series of chemotherapy . The professional health workers involved in this research are nurse educators and practitioners with expertise in oncology and women's health and gynecology specialists.

The FoRSHE-X pilot intervention study began with the development of various learning media, both in the form of videos and infographics which will be uploaded to YouTube and Instagram which can be accessed by participants in this study using smartphones, tablets or computers. The educational media that have been developed are socialized to the control group before the telecoaching activity begins. The educational media that have been developed include: one e-book (Overcoming Various Side Effects due to Chemotherapy for Cancer Patients), four videos (management of chemotherapy side effects, mindfulness therapy, management of sexual problems during cancer therapy, and learning from cancer survivors) and 14 infographics (hair loss, fatigue, anemia, nausea, bleeding, anxiety, vomiting, pain, vaginal dryness, pain during sexual intercourse, sexual communication with husband, how to generate desire for sexual intercourse, and dry skin) provided as a pre-education media telecoaching.

Methodology

The design of this pilot study is *a mixed -methods* prospective longitudinal study (combining a prospective longitudinal intervention study and a longitudinal qualitative study) which seeks to assess the feasibility and effectiveness of the FoRSHE-X digital intervention package in gynecological cancer survivors undergoing chemotherapy. Researchers will implement the FoRSHE-X digital intervention package in two activity stages, where phase I lasts for 6 weeks and phase II lasts for 4 weeks (see table 1). Researchers will start this

intervention activity in September 2023, and it is hoped that this activity will end in January 2023.

Table 1. Stages of implementation of the FoRSHE-X digital package

Phase	Week	Activity	Indicators
Working Phase I	I	Pre-test, digital education , evaluation 1	Knowledge effect side (physical , mental, sexual)
	II	Telecoaching session 1	Handling worry
	III	Telecoaching session 2	Management effect side (physical)
	IV	Telecoaching session 3	Quality connection sexual
	V	Telecoaching session 4	Efficacy self
	VI	Evaluation 2	
Working Phase II	VII	Telecoaching session 5 *(on demand)*	Handling effect side effects and management
	VIII	Telecoaching session 6 *(on demand)*	
	IX	Telecoaching session 7 *(on demand)*	
	X	Evaluation 3	

Successful clinical experimental pilot study *(pre-post evaluation design)* This will be measured through pre and post-tests regarding levels of anxiety, self-efficacy, quality sexual life , and knowledge and management . Meanwhile, qualitative data collection using in-depth semi-structured interviews was carried out to evaluate the feasibility, acceptability and satisfaction of participants in participating in FoRSHE-X digital education and *tele-coaching activities* . (See Chart 1.)

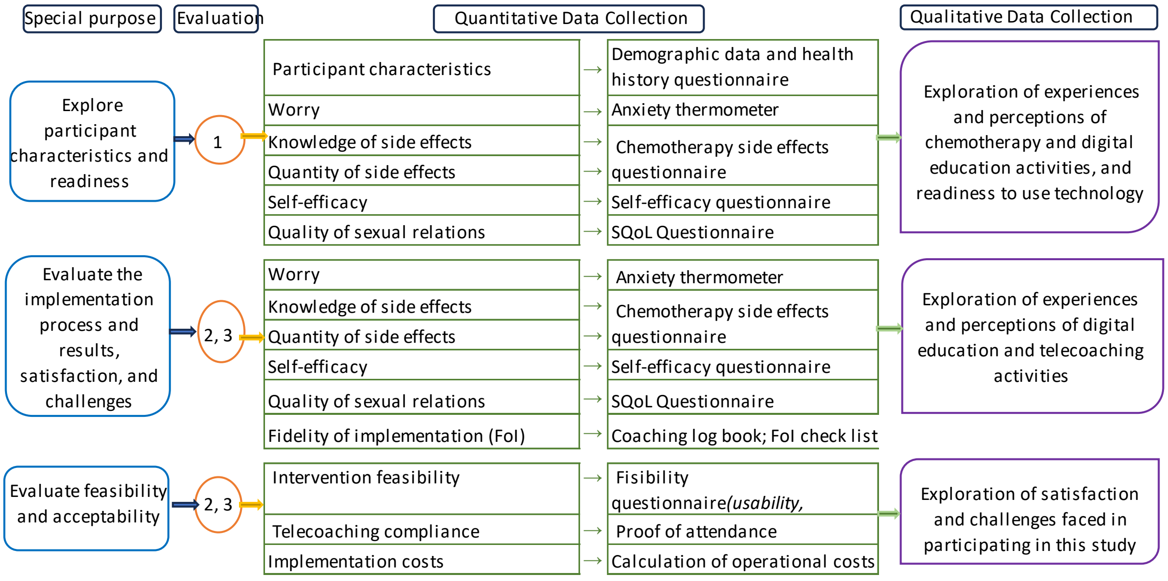

Chart 1. Overview of specific objectives and data collection process.

Research Instrument

1. Demographic data and health history questionnaire.
2. Anxiety thermometer
3. Chemotherapy side effects questionnaire (knowledge and quantity)
4. Self-efficacy questionnaire
5. *Sexual Quality of Life* Questionnaire (SQoL-F)
6. Coaching log book
7. Fidelity of Implementation (FoI) checklist.
8. Fisibility questionnaire
9. Proof of attendance in digital education and telecoaching activities.
10. Record of operational cost calculations for FoRSHE-X digital interventions.

Population and Sample/participants

The population in this study were all gynecological cancer survivors who were undergoing chemotherapy at Dharmais Cancer Hospital. The sample in the quantitative study was 30 participants who were gynecological cancer survivors who were in the early stages of the cycle Chemo therapy was carried out at Dharmais Cancer Hospital and recruited using non-randomized *consecutive sampling.* This sampling technique determines the samples that will be involved in this research based on their presence at the research location. fulfillment of inclusion criteria, and involvement of all samples until the specified number of samples is met (Thewees, et al., 2018). Determining a minimum sample size of 30 participants is determined based on the assumption that the main purpose of conducting a pilot study is not to test a hypothesis, so the sample size in a pilot study is often not calculated using a certain formula. Some researchers use a sample size of 30 people per group in the research activities they carry out. (I, 2017). On the other hand, in-depth interview activities will involve 20 participants who are currently or have taken part in FoRSHE-X digital education and *tele-coaching* .

Criteria inclusion Participants in this research included:

1. Have a smartphone personal
2. Want to learn or Already able to use online media
3. Willing to follow the activity process implementation until finished .
4. Able to communicate well in Indonesian .

Criteria exclusion :

1. Survivor experiencing cancer recurrence or Already undergo chemotherapy previously .
2. Survivor cancer that is not married or not own partner sexual .

Research Flow

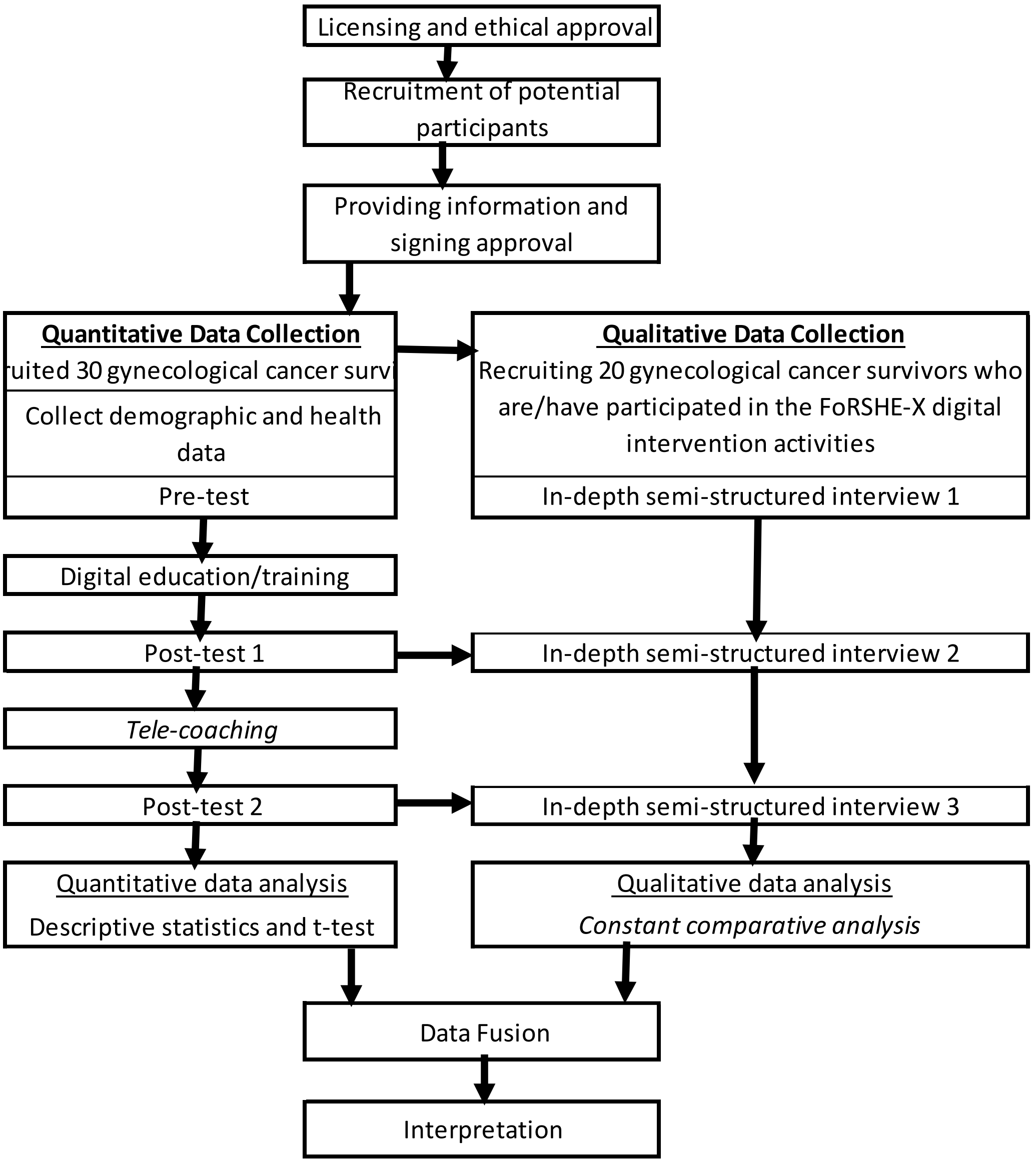

Chart 2. Research Flow

Research Ethics

Before research activities are carried out, participants are given an explanation of the activities to be carried out and participant rights, as explained in the Information Sheet . Participants are given the opportunity to ask questions that are unclear and have the right to refuse to participate in this research activity. Next, if the participant agrees to become a participant, he or she will sign the Consent Form.

Compensation

Participants will be given a data package of IDR 100,000/month for carrying out 2.5 months of research activities. Apart from that, funds to replace the time used to take part in this study, amounting to IDR 100,000, will also be given to each participant at the end of the

implementation of the FoRSHE-X digital package. Meanwhile, some participants will also be interviewed in depth after providing digital education, at the end of working phase I, and at the end of working phase II, for IDR. 100,000,- as a replacement for transport and time used during the interview process. Interviews can be conducted offline or online, depending on the participant's wishes.

Attachment:

1. Information sheet and research approval form.
2. Log Book Coaching
3. SOP for coaching activities
4. SAP and Digital Education Activity Schedule

References

Afiyanti, Y., Gayatri, D., Besral, B., & Haryani, H. (2019). Unmet supportive care needs of Indonesian gynecological cancer survivors. *Enferm Clin, 29 Suppl 2* , 869-873. doi: https://doi.org/10.1016/j.enfcli.2019.04.131

Afiyanti, Y., Milanti, A., & Putri, RH (2018). Supportive care needs in predicting the quality of life among gynecological cancer patients. *Can Oncol Nurs J, 28* (1), 22-29. doi:10.5737/236880762812229

Afiyanti, Y., Setyowati, Milanti, A., & Young, A. (2020). 'Finally, I get to a climax': the experiences of sexual relationships after a psychosexual intervention for Indonesian cervical cancer survivors and their husbands. *Journal of Psychosocial Oncology, 38* (3), 293-309. doi:10.1080/07347332.2020.1720052

Barakat, S., Boehmer, K., Abdelrahim, M., Ahn, S., Al-Khateeb, A.A., Villalobos, N. Á., . . . Murad, M. H. (2017). Does Health Coaching Grow Capacity in Cancer Survivors? A Systematic Review. *Population Health Management, 21* (1), 63-81. doi:10.1089/pop.2017.0040

Beesley, V. L., Alemayehu, C., & Webb, P. M. (2018). A systematic literature review of the prevalence of and risk factors for supportive care needs among women with gynecological cancer and their caregivers. *Supportive Care in Cancer, 26* (3), 701-710. doi:10.1007/s00520-017-3971-6

Boa, R., & Grenman, S. (2018). Psychosexual health in gynecologic cancer. *Int J Gynaecol Obstet, 143 Suppl 2* , 147-152. doi:10.1002/ijgo.12623

Galica, J., Giroux, J., Francis, J.-A., & Maheu, C. (2020). Coping with fear of cancer recurrence among ovarian cancer survivors living in small urban and rural settings: A qualitative descriptive study. *European Journal of Oncology Nursing, 44* , 101705. doi: https://doi.org/10.1016/j.ejon.2019.101705

Galica, J., Zwaal, C., Kennedy, E., Asmis, T., Cho, C., Ginty, A., & Govindarajan, A. (2022). Models of Follow-Up Care and Secondary Prevention Measures for Survivors of Colorectal Cancer: Evidence-Based Guidelines and Systematic Review. *29* (2), 439-454.

In, J. (2017) Introduction of a pilot study **,** *Korean J Anesthesiol ;* 70(6):601-605. **https://doi.org/10.4097/kjae.2017.70.6.601**

Lokich, E. (2019). Gynecologic Cancer Survivorship. *Obstet Gynecol Clin North Am, 46* (1), 165-178. doi: https://doi.org/10.1016/j.ogc.2018.10.002

McCallum, M., Jolicoeur, L., Lefebvre, M., Babchishin, L.K., Robert-Chauret, S., Le, T., & Lebel, S. (2014). Supportive care needs after gynecologic cancer: where does sexual health fit in? *Oncol Nurs Forum, 41* (3), 297-306. doi: http://dx.doi.org/10.1188/14.ONF.297-306

Mlakar, I., Lin, S., Aleksandraviča, I., Arcimoviča, K., Eglītis, J., Leja, M., . . . Smrke, U. (2021). Patient-centered Survivorship care plan after Cancer treatments based on Big Data and Artificial Intelligence technologies (PERSIST): a multicenter study protocol to evaluate the efficacy of digital tools supporting cancer survivors. *BMC Medical Informatics and Decision Making, 21* (1), 243. doi:10.1186/s12911-021-01603-w

Nápoles, A.M., Santoyo-Olsson, J., Chacón, L., Stewart, A.L., Dixit, N., & Ortiz, C. (2019). Feasibility of a Mobile Phone App and Telephone Coaching Survivorship Care Planning Program Among Spanish-Speaking Breast Cancer Survivors. *JMIR Cancer, 5* (2), e13543. doi:10.2196/13543

Nekhlyudov, L., Mollica, M.A., Jacobsen, P.B., Mayer, D.K., Shulman, L.N., & Geiger, A.M. (2019). Developing a Quality of Cancer Survivorship Care Framework: Implications for Clinical Care, Research, and Policy. *JNCI: Journal of the National Cancer Institute, 111* (11), 1120-1130. doi:10.1093/jnci/djz089 %J JNCI: Journal of the National Cancer Institute

Schlumbrecht, M., Sun, C., Huang, M., Milbourne, A., & Bodurka, D. (2018). Gynecologic cancer survivor preferences for long-term surveillance. *BMC Cancer, 18* (1), 375. doi:10.1186/s12885-018-4313-x

Sung, H., Ferlay, J., Siegel, R.L., Laversanne, M., Soerjomataram, I., Jemal, A., & Bray, F. (2021). Global Cancer Statistics 2020: GLOBOCAN Estimates of Incidence and Mortality Worldwide for 36 Cancers in 185 Countries. *CA Cancer J Clin, 71* (3), 209-249. doi:10.3322/caac.21660

Thewes B, Rietjens JAC, van den Berg SW, Compen FR, Abrahams H, Poort H, ……… Prins JB. (2018). One way or another: The opportunities and pitfalls of self-referral and consecutive sampling as recruitment strategies for psycho-oncology intervention trials. *Psychooncology;* 27(8):2056-2059. doi: 10.1002/pon.4780.

Wagner, LI, Tooze, JA, Hall, D.L., Levine, B.J., Beaumont, J., Duffecy, J., . . . Cella, D. (2021). Targeted eHealth Intervention to Reduce Breast Cancer Survivors' Fear of Recurrence: Results From the Fortitude Randomized Trial. *JNCI: Journal of the National Cancer Institute, 113* (11), 1495-1505. doi:10.1093/jnci/djab100 %J JNCI: Journal of the National Cancer Institute

PROTOKOL PENELITIAN

PENGEMBANGAN INTERVENSI DIGITAL (EDUKASI DAN *TELE-COACHING)* FORSHE-X UNTUK MENINGKATKAN KUALITAS HIDUP PASIEN KANKER GINEKOLOGI: STUDI PILOT DAN FISIBILITAS

Peneliti:

6. **Prof. Dr. Yati Afiyanti, S.Kp., MN**
7. **Dyah Juliastuti, S.Kp., M.Sc., M.Kep., Sp.Mat, Ph.D**
8. **Ns. Ariesta Milanti, S.Kep., MHC, Ph.D**
9. **Ns. Lina Anisa Nasution, S.Kep., M.Kep.**
10. **Ns. Aprilia Dian Prawesti, S.Kep**

Fakultas Ilmu Keperawatan
Universitas Indonesia
Tahun 2023

Judul (*Title*)
Pengembangan Intervensi Digital (Edukasi dan *Tele-coaching*) FoRSHE-X untuk meningkatkan Kualitas Hidup Pasien Kanker Ginekologi: Studi Pilot dan Fisibilitas

Abstrak (*Abstract)*
Kanker ginekologi merupakan salah satu penyakit tidak menular yang memiliki prevalensi kejadian dan kematian tertinggi pada perempuan Indonesia. Perburukan kondisi akibat kanker ginekologi dapat meningkatkan biaya pengobatan dan menurunkan kualitas hidup penyintas kanker. Hal ini dapat dicegah dengan pemberian intervensi keperawatan berbasis teknologi digital yang terbukti meningkatkan kesehatan fisik, mental, dan seksual penyintas kanker di berbagai negara. Penelitian ini bertujuan untuk mengembangkan fisibilitas dan akseptabilitas serta mengevaluasi efek intervensi paket edukasi digital dan *tele-coaching* FoRSHE-X (*Fight on Recurring anxiety, Self-efficacy, Health Effects, and seXual deprivation*) pada penyintas kanker ginekologi yang sedang menjalankan kemoterapi. Desain studi pilot ini adalah penelitian prospective longitudinal *mixed-methods* yang mengevaluasi proses dan hasil implementasi, serta fisibilitas intervensi digital FoRSHE-X dalam suatu studi yang mengintegrasikan pemanfaatan media teknologi, edukasi digital, dan konsep *tele-coaching* dalam meningkatkan kemandirian penyintas kanker ginekologi mengatasi berbagai efek samping kemoterapi dan meningkatkan keberlanjutan terapi kanker.

Kata Kunci (Keywords)
efikasi diri, efek samping, FoRSHE-X, kanker ginekologi, kecemasan, kualitas hubungan seksual, penyintas, perawatan diri, pilot, fisibilitas

Latar Belakang *(Background)*
Penyebab kematian tertinggi pada perempuan adalah kanker (Sung et al., 2021). Jenis kanker terbanyak pada perempuan adalah kanker payudara, kanker serviks, kanker ovarium dan kanker korpus uterina (Sung et al., 2021). Besaran kasus tersebut tentu saja seiring dengan besarnya perhatian yang dibutuhkan oleh penyintas kanker terutama terkait angka ketahanan hidup (*survivor rate*) dan kualitas hidup penyintas yang menjadi isu dalam *Sustainable Development Goals* (SDGs) pada sektor kesehatan. Perawatan pada penyintas penyakit tersebut termasuk dalam sembilan program pokok yang dicanangkan oleh Kementerian Kesehatan (Afiyanti, Gayatri, Besral, & Haryani, 2019; Afiyanti, Milanti, & Putri, 2018).

Penyintas kanker ginekologi akan memiliki prognosis yang lebih baik untuk mempertahankan hidupnya (*survivor rate)* jika mereka mampu meyelesaikan rangkaian terapi yang telah direncanakan dan memahami manajemen kesehatan di masa dan paska terapi (Mlakar et al., 2021; Schlumbrecht, Sun, Huang, Milbourne, & Bodurka, 2018). Saat menjalankan terapi, penyintas kanker ginekologi memiliki kebutuhan multidimensional yang jika tidak diantisipasi akan dapat mengakibatkan penghentian terapi, kekambuhan, bahkan kematian (Galica, Giroux, Francis, & Maheu, 2020; Nekhlyudov et al., 2019). Kebutuhan tersebut berhubungan dengan pencegahan risiko kekambuhan, gaya hidup sehat, penatalaksanaan gejala fisik akibat perjalanan penyakit maupun efek terapi yang bersifat jangka panjang dan masalah psikososial dan seksual, serta kebutuhan informasional

mengenai perawatan diri mandiri (Beesley, Alemayehu, & Webb, 2018; Galica et al., 2022; Schlumbrecht et al., 2018).

Beberapa studi terdahulu di Indonesia menunjukkan bahwa penyintas kanker ginekologi melaporkan keterbatasan waktu dan kesempatan untuk bertanya dan berdikusi dengan tim kesehatan, khususnya tim keperawatan, terkait permasalahan fisik, mental, sosial, dan seksual yang mereka hadapi disaat menjalani terapi kanker (Afiyanti et al., 2019; Afiyanti, Setyowati, Milanti, & Young, 2020; Boa & Grenman, 2018). Hambatan dalam mengkomunikasikan permasalahan kesehatan dan hal-hal yang terkait lainnya dapat mengakibatkan terjadinya henti terapi, keterlambatan dalam mencari pertolongan, dan ketidakmampuan perempuan penyintas dalam mengantisipasi kekambuhan dan konflik dengan pasangan seksualnya (Lokich, 2019; McCallum et al., 2014). Pemanfaatan teknologi digital telah terbukti efektif dalam meningkatkan kesehatan fisik, mental, dan seksual penyintas berbagai jenis kanker ginekologi di beberapa negara (Barakat et al., 2017; Nápoles et al., 2019; Wagner et al., 2021).

Masalah kesehatan dan kebutuhan perawatan pada penyintas kanker ginekologi telah dieksplorasi oleh berbagai riset terdahulu, namun, intervensi keperawatan berbasis internet bagi penyintas kanker belum secara intensif dikembangkan dalam tatanan pelayanan keperawatan di Indonesia dan belum ditemukan metode intervensi yang bisa menjangkau pelayanan keperawatan yang lebih luas dan sensitive terhadap budaya Indonesia. Pemanfaatan tele-edukasi dan *tele-coaching* berkelanjutan diharapkan dapat mendukung efikasi diri penyintas kanker ginekologi dalam mencegah dan mengatasi masalah yang dapat menghambat mereka melanjutkan terapi dan mempersiapkan mereka mengahadapi periode paska terapi. Intervensi ini dapat menghubungkan pasien dengan tim keperawatan tanpa dibatasi ruang dan waktu. Pemanfaatan digital teknologi diyakini dapat memperluas jangkauan pelayanan keperawatan dan meningkatkan partisipasi pasien dalam perawatan dirinya sendiri secara berkelanjutan. Intervensi digital FoRSHE-X berpotensi memenuhi kebutuhan kesehatan penyintas kanker ginekologi yang sedang menjalani kemoterapi dan meningkatkan ketahanan hidup yang capaiannya masih rendah di Indonesia serta mengurangi beban keluarga dan Negara akibat biaya pengobatan kanker.

Tujuan Umum Riset (*Research General Aim*)

Studi pilot ini bertujuan untuk mengembangkan fisibilitas dan akseptabilitas serta mengevaluasi efek intervensi paket edukasi digital dan *tele-coaching* FoRSHE-X (*Fight on Recurring anxiety, Self-efficacy, Health Effects, and seXual deprivation*) pada penyintas kanker ginekologi yang sedang menjalankan kemoterapi.

Tujuan Khusus Riset (*Research Objectives*)

4. Menggali karakteristik umum (usia, pendidikan terakhir, pekerjaan, marital status) dan riwayat kesehatan (lama terdiagnosa kanker, stadium kanker, jenis dan jumlah sesi terapi kanker yang sudah dijalani), serta kesiapan penyintas kanker ginekologi yang sedang menjalankan kemoterapi mengikuti kegiatan .

5. Mengevaluasi proses dan hasil implementasi, serta kepuasan dan tantangan bagi penyintas kanker ginekologi yang sedang menjalani kemoterapi dalam memanfaatkan paket intervensi digital FoRSHE-X.
6. Mengevaluasi feasibilitas dan akseptabilitas paket intervensi digital FoRSHE-X dalam meningkatkan kualitas hidup penyintas kanker ginekologi.

Hipotesis dalam penelitian ini:

5. Terdapat penurunan tingkat kecemasan penyintas kanker ginekologi yang sedang menjalankan kemoterapi setelah pemberian intervensi paket FoRSHE-X
6. Terdapat peningkatan efikasi diri penyintas kanker ginekologi yang sedang menjalankan kemoterapi.etelah pemberian intervensi paket FoRSHE-X
7. Terdapat peningkatan pengetahuan dan penurunan kuantitas efek samping pada penyintas kanker ginekologi yang sedang menjalankan kemoterapi setelah pemberian intervensi paket FoRSHE-X
8. Terdapat peningkatan kualitas kehidupan seksual yang lebih besar pada penyintas kanker ginekologi yang sedang menjalankan kemoterapi.setelah pemberian intervensi paket FoRSHE-X

Intervensi Digital FoRSHE-X

Prototif paket digital (edukasi dan tele-coach) ForSHE-X adalah suatu intervensi digital yang mengkombinasikasi edukasi berbasis sosial media (instagram dan zoom) dan *tele-coaching* (via wahtsapp video call atau zoom) untuk mendukung kebutuhan perawatan penyintas kanker ginekologi yang sensitif budaya Indonesia. Intervensi-intervensi dalam paket FoRSHE-X difokuskan untuk menurunkan ketegangan kecemasan akan proses terapi, meningkatkan pengetahuan dan kuantitas efek samping, meningkatkan efikasi diri, dan meningkatkan kualitas kehidupan seksual para penyintas kanker ginekologi saat menjalanin rangkaian kemoterapi. Tenaga kesehatan professional yang terlibat dalam penelitian ini adalah perawat edukator dan pelaksana dengan keahlian onkologi dan kesehatan perempuan dan dokter spesialis ginekologi.

Studi pilot intervensi FoRSHE-X ini diawali dengan pengembangan berbagai media pembelajaran, baik berupa video maupun yang berbentuk infografis yang akan di upload dalam youtube dan instagram yang dapat diakses oleh partisipan pada penelitian ini dengan menggunakan smartphone, tablet, ataupun komputer. Media-media edukasi yang telah dikembangkan disosialisasikan kepada kelompok kontrol sebelum kegiatan telecoaching dimulai. Adapun media edukasi yang telah dikembangkan meliputi: sartu e-book (Mengatasi Berbagai Efeksamping akibat Kemoterapi untuk Pasien Kanker), empat video (manajemen efek samping kemoterapi, terapi mindfulness, manajemen masalah seksual selama menjalani terapi kanker, dan belajar dari penyintas kanker) dan 14 infografis (rambut rontok, kelelahan, anemia, mual, perdarahan, cemas, muntah, nyeri, vagina kering, nyeri saat berhubungan seksual, komunikasi seksual dengan suami, cara memunculkan hasrat melakukan hubungan seksual, dan kulit kering) yang disediakan sebagai media edukasi pra-telecoaching.

Metodologi

Desain studi pilot ini adalah *mixed-methods* prospektif longitudinal (mengkombinasikan studi intervensi prospektif longitudinal dan studi kualitatif longitudinal) yang berupaya mengkaji fisibilitas dan efektivitas paket intervensi digital FoRSHE-X pada penyintas kanker ginekologi yang sedang menjalankan kemoterapi. Peneliti akan mengimplementasikan paket intervensi digital FoRSHE-X dalam dua tahap kegiatan, dimana tahap I berlangsung selama 6 minggu dan tahap II berlangsung selama 4 minggu (lihat tabel 1). Peneliti akan memulai kegiatan intervensi ini pada bulan September 2023, dan diharapkan kegiatan ini akan berakhir pada bulan Januari 2023.

Tabel 1. Tahapan implementasi paket digital FoRSHE-X

Phase	Week	Activity	Indicator
Working Phase I	I	Pre-test, edukasi digital, evaluasi 1	Pengetahuan efek samping (fisik, mental, seksual)
	II	Telecoaching sesi 1	Penanganan kecemasan
	III	Telecoaching sesi 2	Penatalaksanaan efek samping (fisik)
	IV	Telecoaching sesi 3	Kualitas hubungan seksual
	V	Telecoaching sesi 4	Efikasi diri
	VI	Evaluasi 2	
Working Phase II	VII	Telecoaching sesi 5 *(on demand)*	Penanganan efek samping dan penatalaksanaannya
	VIII	Telecoaching sesi 6 *(on demand)*	
	IX	Telecoaching sesi 7 *(on demand)*	
	X	Evaluasi 3	

Keberhasilan studi pilot eksperimental klinik *(pre-post evaluation design)* ini akan diukur melalui pre dan post-test tentang tingkat kecemasan, efikasi diri, kualitas kehidupan seksual, dan pengetahuan serta penatalaksanaannya. Sementara itu, pengumpulan data kualitatif dengan wawancara mendalam semi-struktur dilakukan untuk mengevaluasi fisibilitas, akseptabiliti, dan kepuasan partisipan dalam mengikuti kegiatan edukasi digital dan *tele-coaching* FoRSHE-X. (Lihat Bagan 1.)

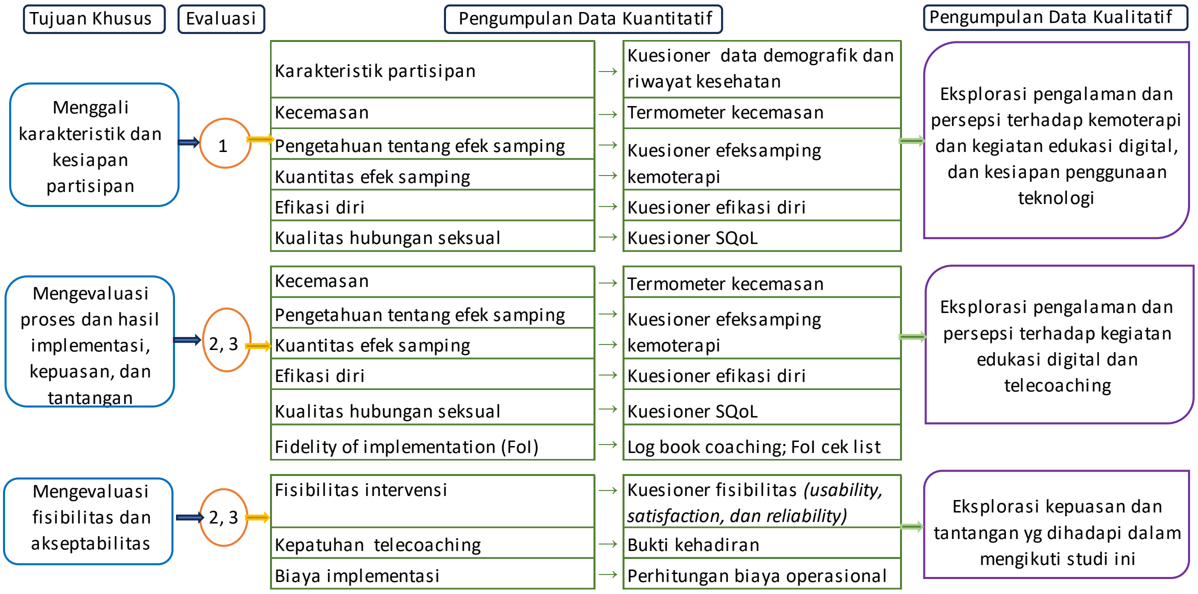

Bagan 1. Tinjauan tujuan khusus dan proses pengumpulan data.

Instrumen Penelitian

11. Kuesioner data demografik dan riwayat kesehatan.
12. Termometer kecemasan
13. Kuesioner efek samping kemoterapi (pengetahuan dan kuantitas)
14. Kuesioner efikasi diri
15. Kuesioner *Sexual Quality of Life* Perempuan (SQoL-F)
16. Log book Coaching
17. Daftar cek Fidelity of Implementation (FoI).
18. Kuesioner fisibilitas
19. Bukti kehadiran dalam kegiatan edukasi digital dan telecoaching.
20. Catatan perhitungan biaya operasional intervensi digital FoRSHE-X.

Pupulasi dan Sampel/partisipan

Populasi pada penelitian ini adalah semua penyintas kanker ginekologi yang sedang menjalani kemoterapi di RS Kanker Dharmais. Sampel pada studi kuantitatif adalah 30 partisipan yang merupakan penyintas kanker ginekologi yang berada pada tahap awal siklus kemoterapi yang dilakukan di RS Kanker Dharmais dan direkrut secara non-randomisasi *consecutive sampling*. Tehnik sampling ini menentukan sampel yang akan dilibatkan dalam penelitian ini berdasarkan kehadirannya di lokasi penelitian. pemenuhan kriteria inklusi, dan pelibatan semua sampel sampai jumlah sample yang ditetapkan dapat terpenuhi (Thewees, et al., 2018). Penentuan jumlah sample minimal 30 partsipan ditentukan berdasarkan asumsi bahwa tujuan utama dilakukannya pilot studi adalah bukan untuk menguji hipotesis, sehingga jumlah sampel pada pilot studi sering tidak dihitung menggunakan suatu rumus tertentu Beberapa peneliti menggunakan jumlah sample 30 orang per grup dalam kegiatan penelitian yang mereka lakukan. (I, 2017). Di lain sisi, kegiatan wawancara mendalam akan melibatkan

20 orang partsipan yang sedang atau telah mengikuti ke edukasi digital dan *tele-coaching* FoRSHE-X.

Kriteria inklusi partisipan pada penelitian ini meliputi:

5. Memiliki smartphone pribadi
6. Mau belajar atau sudah mampu menggunakan media online
7. Bersedia mengikuti proses kegiatan implementasi sampai selesai.
8. Mampu berkomunikasi dengan baik dalam bahasa Indonesia.

Kriteria eksklusi:

3. Penyintas kanker yang mengalami kekambuhan atau sudah menjalani kemoterapi sebelumnya.
4. Penyintas kanker yang tidak menikah dan tidak memiliki pasangan seksual.

Alur Penelitian

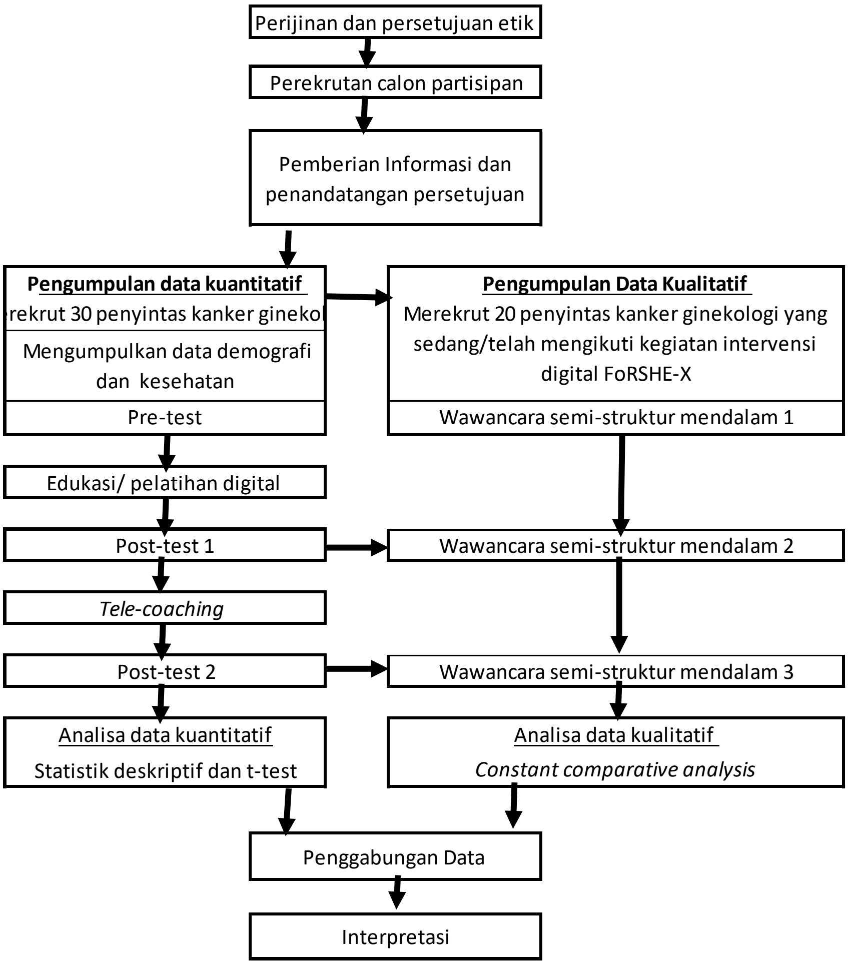

Bagan 2. Alur Penelitian

Etik Penelitian

Sebelum kegiatan penelitian dilakukan, partisipan diberikan penjelasan tentang kegiatan yang akan dilakukan dan hak-hak partisipan, seperti yang dijelaskan dalam Lembar

Informasi. Partisipan diberikan kesempatan untuk menanyakan hal-hal yang kurang jelas dan berhak untuk monalak berpartisipasi dalam kegiatan penelitian ini. Selanjutnya, jika partisipan setuju untuk menjadi partisipan maka ia akan menenandatangani Lembar Persetujuan.

Kompensasi

Partisipan akan diberikan paket data Rp, 100.000,-/ bulan untuk pelaksanaan 2,5 bulan kegiatan penelitian. Selain itu, dana pengganti waktu yang digunakan untuk mengikuti studi ini, sebesar Rp, 100.000, juga akan diberikan kepada setiap partisipan diakhir implementasi paket digital FoRSHE-X. Sementara itu, sebagian partisipan juga akan diwawancarai secara mendalam paska pemberian edukasi digital, diakhir working phase I, dan diakhir working phase II, sebesar Rp. 100.000,- sebagai pengganti transport dan waktu yang digunakan selama proses wawancara. Wawancara dapat dilakukan secara luring atau daring, disesuaikan dengan keinginan partisipann.

Lampiran:

5. Lembar informasi dan lembar persetujuan penelitian.
6. Log Book Coaching
7. SOP kegiatan coaching
8. SAP dan Jadwal Kegiatan Edukasi Digital

References

Afiyanti, Y., Gayatri, D., Besral, B., & Haryani, H. (2019). Unmet supportive care needs of Indonesian gynecological cancer survivors. *Enferm Clin, 29 Suppl 2*, 869-873. doi:https://doi.org/10.1016/j.enfcli.2019.04.131

Afiyanti, Y., Milanti, A., & Putri, R. H. (2018). Supportive care needs in predicting the quality of life among gynecological cancer patients. *Can Oncol Nurs J, 28*(1), 22-29. doi:10.5737/236880762812229

Afiyanti, Y., Setyowati, Milanti, A., & Young, A. (2020). 'Finally, I get to a climax': the experiences of sexual relationships after a psychosexual intervention for Indonesian cervical cancer survivors and the husbands. *Journal of Psychosocial Oncology, 38*(3), 293-309. doi:10.1080/07347332.2020.1720052

Barakat, S., Boehmer, K., Abdelrahim, M., Ahn, S., Al-Khateeb, A. A., Villalobos, N. Á., . . . Murad, M. H. (2017). Does Health Coaching Grow Capacity in Cancer Survivors? A Systematic Review. *Population Health Management, 21*(1), 63-81. doi:10.1089/pop.2017.0040

Beesley, V. L., Alemayehu, C., & Webb, P. M. (2018). A systematic literature review of the prevalence of and risk factors for supportive care needs among women with gynaecological cancer and their caregivers. *Supportive Care in Cancer, 26*(3), 701-710. doi:10.1007/s00520-017-3971-6

Boa, R., & Grenman, S. (2018). Psychosexual health in gynecologic cancer. *Int J Gynaecol Obstet, 143 Suppl 2*, 147-152. doi:10.1002/ijgo.12623

Galica, J., Giroux, J., Francis, J.-A., & Maheu, C. (2020). Coping with fear of cancer recurrence among ovarian cancer survivors living in small urban and rural settings: A qualitative descriptive study. *European Journal of Oncology Nursing, 44*, 101705. doi:https://doi.org/10.1016/j.ejon.2019.101705

Galica, J., Zwaal, C., Kennedy, E., Asmis, T., Cho, C., Ginty, A., & Govindarajan, A. (2022). Models of Follow-Up Care and Secondary Prevention Measures for Survivors of Colorectal Cancer: Evidence-Based Guidelines and Systematic Review. *29*(2), 439-454.

In, J. (2017) Introduction of a pilot study, *Korean J Anesthesiol;*70(6):601-605. **https://doi.org/10.4097/kjae.2017.70.6.601**

Lokich, E. (2019). Gynecologic Cancer Survivorship. *Obstet Gynecol Clin North Am, 46*(1), 165-178. doi:https://doi.org/10.1016/j.ogc.2018.10.002

McCallum, M., Jolicoeur, L., Lefebvre, M., Babchishin, L. K., Robert-Chauret, S., Le, T., & Lebel, S. (2014). Supportive care needs after gynecologic cancer: where does sexual health fit in? *Oncol Nurs Forum, 41*(3), 297-306. doi:http://dx.doi.org/10.1188/14.ONF.297-306

Mlakar, I., Lin, S., Aleksandraviča, I., Arcimoviča, K., Eglītis, J., Leja, M., . . . Smrke, U. (2021). Patients-centered SurvivorShIp care plan after Cancer treatments based on Big Data and Artificial Intelligence technologies (PERSIST): a multicenter study protocol to evaluate efficacy of digital tools supporting cancer survivors. *BMC Medical Informatics and Decision Making, 21*(1), 243. doi:10.1186/s12911-021-01603-w

Nápoles, A. M., Santoyo-Olsson, J., Chacón, L., Stewart, A. L., Dixit, N., & Ortiz, C. (2019). Feasibility of a Mobile Phone App and Telephone Coaching Survivorship Care Planning Program Among Spanish-Speaking Breast Cancer Survivors. *JMIR Cancer, 5*(2), e13543. doi:10.2196/13543

Nekhlyudov, L., Mollica, M. A., Jacobsen, P. B., Mayer, D. K., Shulman, L. N., & Geiger, A. M. (2019). Developing a Quality of Cancer Survivorship Care Framework: Implications for Clinical Care, Research, and Policy. *JNCI: Journal of the National Cancer Institute, 111*(11), 1120-1130. doi:10.1093/jnci/djz089 %J JNCI: Journal of the National Cancer Institute

Schlumbrecht, M., Sun, C., Huang, M., Milbourne, A., & Bodurka, D. (2018). Gynecologic cancer survivor preferences for long-term surveillance. *BMC Cancer, 18*(1), 375. doi:10.1186/s12885-018-4313-x

Sung, H., Ferlay, J., Siegel, R. L., Laversanne, M., Soerjomataram, I., Jemal, A., & Bray, F. (2021). Global Cancer Statistics 2020: GLOBOCAN Estimates of Incidence and Mortality Worldwide for 36 Cancers in 185 Countries. *CA Cancer J Clin, 71*(3), 209-249. doi:10.3322/caac.21660

Thewes B, Rietjens JAC, van den Berg SW, Compen FR, Abrahams H, Poort H,………Prins JB. (2018). One way or another: The opportunities and pitfalls of self-referral and consecutive sampling as recruitment strategies for psycho-oncology intervention trials. *Psychooncology;* 27(8):2056-2059. doi: 10.1002/pon.4780.

Wagner, L. I., Tooze, J. A., Hall, D. L., Levine, B. J., Beaumont, J., Duffecy, J., . . . Cella, D. (2021). Targeted eHealth Intervention to Reduce Breast Cancer Survivors' Fear of Recurrence: Results From the FoRtitude Randomized Trial. *JNCI: Journal of the National Cancer Institute, 113*(11), 1495-1505. doi:10.1093/jnci/djab100 %J JNCI: Journal of the National Cancer Institute
