# Supplementary material for: FoRSHE-X digital health intervention to improve the quality of life during chemotherapy among gynecological cancer survivors in Indonesia: A protocol for a pilot and feasibility study
Source: PLoS One. 2024 Dec 18;19(12):e0299901. doi: 10.1371/journal.pone.0299901 (PMC11654957; doi:10.1371/journal.pone.0299901)
Supplement: S1 Text — (PDF) [file pone.0299901.s003.pdf]

PARTICIPANT INFORMATION SHEET

The undersigned below, I:

Name: **Prof. Dr. Afiyanti, S.Kp., MN**
Faculty of Nursing, University of Indonesia
No. Contact: 0813-1594-3320

ask you to be a respondent in my research which title is "Development of the FoRSHE-X Tele-coaching Intervention to improve the Quality of Life of Gynecological Cancer Patients: Pilot Study and Feasibility". This study aims to develop and evaluate the tele-coaching intervention FoRSHE-X (Fight on Stress, Health Effects, and Sexual Deprivation) in improving the quality of life of gynecological cancer survivors who are undergoing chemotherapy/radiotherapy.

On this occasion, I ask you to participate in this research. You will fill out a questionnaire which measures sociodemographic data, distress, self-efficacy, and quality of sexual life. To complete the questionnaire, it takes approximately 15 minutes. Next, you will take part in a series of tele-education and tele-coaching activities via Zoom or WhatsApp meetings individually, as well as access information about treatment management during chemotherapy via social media Instagram and YouTube. Online mentoring activities will be carried out every week during the 10 week study period. Apart from filling out the questionnaire, several participants will be involved in in-depth interviews to assess the feasibility study and opportunities for sustainability of the FoRSHE-X program.

We are asking you to share with us some very personal and confidential information, and you may feel uncomfortable of talking about some of the topics. You have the right not to participate in this research without affecting the care they will receive. If during the research you feel uncomfortable, you can withdraw from being a respondent whenever you want. If there is information that you do not understand regarding this research, you can contact the researcher at the telephone number above.

You will receive an internet data plan worth IDR 100,000 per month for the 2.5-month duration of the research activities, a compensation of IDR 100,000 at the end of the FoRSHE-X digital intervention to offset the time spent participating in this study. The participants who will also be interviewed after the provision of digital education and tele-coaching will receive IDR 100,000 as compensation for transportation and time used during the interview process.

The gathered information will be stored securely inside the Chief Investigator cloud. The data that we collect from this research project will be kept private, unless any data without your identity information and have a number or pseudonym to replace your name will be save inside the university library repository.

Thank you for your willingness to participate in this study.

Best regards,

Prof. Dr. Yati Afiyanti, S.Kp, M.N.

INFORMED CONSENT

The undersigned below, I:

Name : ....

Age : ....

States that:

1. I have received an explanation of the research "Development of the FoRSHE-X Tele-coaching Intervention to improve the Quality of Life of Gynecological Cancer Patients: Pilot Study and Feasibility" process of intervention and data collection.

2. I have been given the opportunity to ask questions and get open answers from the researcher.

3. I understand the research procedures to be carried out, the objectives, benefits and possible negative impacts that may occur from the research being carried out.

4. Agree that the data collected can be seen by other people (other than researchers) as long as they do not mention my name.

Based on the considerations above, I hereby agree to voluntarily and without coercion from any party to involve in this study as a participant.

Thus, I made this statement to be used properly.

Jakarta, ____________________

(Full name)
